# MRI-Based Radiomics Ensemble Model for Predicting Radiation Necrosis in Brain Metastasis Patients Treated with Stereotactic Radiosurgery and Immunotherapy

**DOI:** 10.3390/cancers17121974

**Published:** 2025-06-13

**Authors:** Yijun Chen, Corbin Helis, Christina Cramer, Michael Munley, Ariel Raimundo Choi, Josh Tan, Fei Xing, Qing Lyu, Christopher Whitlow, Jeffrey Willey, Michael Chan, Yuming Jiang

**Affiliations:** 1 Department of Radiation Oncology, Wake Forest University School of Medicine, Winston-Salem, NC 27101, USA; yijchen@wakehealth.edu (Y.C.); chelis@wakehealth.edu (C.H.); ccramer@wakehealth.edu (C.C.); mmunley@wakehealth.edu (M.M.); archoi@wakehealth.edu (A.R.C.); jwilley@wakehealth.edu (J.W.); mchan@wakehealth.edu (M.C.); 2 Department of Radiology, Wake Forest University School of Medicine, Winston-Salem, NC 27101, USA; jtan@wakehealth.edu (J.T.); qlyu@wakehealth.edu (Q.L.); cwhitlow@wakehealth.edu (C.W.); 3 Department of Cancer Biology, Wake Forest University School of Medicine, Winston-Salem, NC 27101, USA; fxing@wakehealth.edu

**Keywords:** brain metastasis, radiation necrosis, radiomics, ensemble model, risk prediction

## Abstract

**Background:** Radiation therapy is a primary and cornerstone treatment modality for brain metastasis. However, it can result in complications like necrosis, which may lead to significant neurological deficits. This study aims to develop and validate an ensemble model with radiomics to predict radiation necrosis. **Method:** This study retrospectively collected and analyzed MRI images and clinical information from 209 stereotactic radiosurgery sessions involving 130 patients with brain metastasis. An ensemble model integrating gradient boosting, random forest, decision tree, and support vector machine was developed and validated using selected radiomic features and clinical factors to predict the likelihood of necrosis. The model performance was evaluated and compared with other machine learning algorithms using metrics, including the area under the curve (AUC), sensitivity, specificity, negative predictive value (NPV), and positive predictive value (PPV). SHapley Additive exPlanations (SHAP) analysis and local interpretable model-agnostic explanations (LIME) analysis were applied to explain the model’s prediction. **Results:** The ensemble model achieved strong performance in the validation cohort, with the highest AUC. Compared to individual models and the stacking ensemble model, it consistently outperformed. The model demonstrated superior accuracy, generalizability, and reliability in predicting radiation necrosis. SHAP and LIME were used to interpret a complex predictive model for radiation necrosis. Both analyses highlighted similar significant factors, enhancing our understanding of prediction dynamics. **Conclusions:** The ensemble model using radiomic features exhibited high accuracy and robustness in predicting the occurrence of radiation necrosis. It could serve as a novel and valuable tool to facilitate radiotherapy for patients with brain metastasis.

## Simple Summary

Necrosis is a major complication in brain metastases treated with radiation therapy, highlighting the need to predict the risk associated with each treatment session for earlier prognosis. We developed and validated the first MRI-based radiomics ensemble model to predict necrosis at the lesion level in stereotactic radiosurgery and immunotherapy using data from 209 lesion-based sessions. The model demonstrated robust and reliable performance, outperforming commonly used machine learning algorithms. This approach enables the early identification of high-risk treatment sessions, allowing clinicians to optimize care through computer-assisted strategies aimed at mitigating necrosis risk.

## 1. Introduction

Brain metastasis is the most frequent and feared neurologic complication related to cancer since even small tumors may cause incapacitating neurologic symptoms [1]. Stereotactic radiosurgery (SRS) is now the primary treatment for patients with brain metastases, with potential synergistic effects when combined with certain immunotherapy or targeted therapies [2]. Radiation necrosis is a consequence of radiotherapy for brain metastasis, referring to a delayed and typically progressive and irreversible form of radiation-induced inflammation or injury to normal brain parenchyma [3]. It most commonly occurs at the site of maximum radiation dose [4] and is especially expected in SBS [5]. Thus, predicting radiation necrosis before radiosurgery is crucial for optimizing treatment strategies.

Magnetic resonance imaging (MRI) plays a vital role in predicting radiation necrosis in brain metastasis. Many previous studies have used radiomic features from MRI to distinguish recurrent brain metastasis from radiation necrosis [6,7,8,9,10], detect radiation necrosis [11], and predict radiation response or injury in other carcinomas [12,13], which demonstrates the potential of using MRI-based radiomics to predict radiation necrosis. Ensemble models with radiomics also show success in metastasis detection, classification, and radiosurgery outcome prediction [14,15,16,17]. All these studies show the potential of using an ensemble model with MRI-based radiomics to predict radiation necrosis.

In this study, we aimed to develop an MRI-based radiomics ensemble model to predict radiation necrosis directly. Furthermore, we employed SHapley Additive exPlanations (SHAP) analysis [18,19] and local interpretable model-agnostic explanations (LIME) [20,21,22] analysis to interpret the model’s prediction.

## 2. Materials and Methods

### 2.1. Study Participation

**Figure 1:**
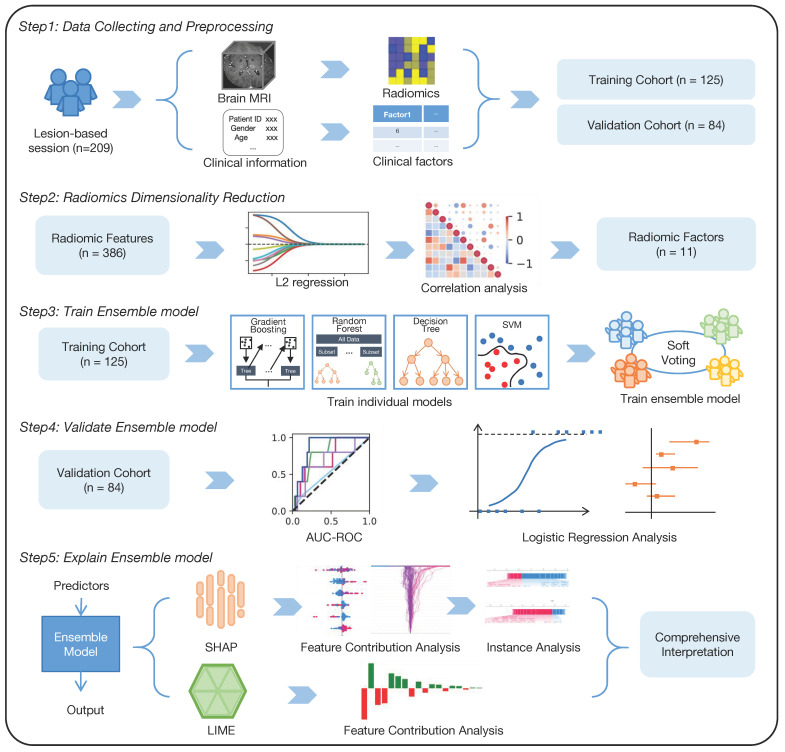
Study design overview. This study involved a retrospective collection and analysis of brain MRI images and clinical data, including oncology information. An ensemble learning model was developed and validated using radiomic features and clinical factors to predict necrosis following radiotherapy for brain metastasis. The model’s performance was assessed using metrics. Model interpretation analyses were employed to explain the model’s predicted values as the sum of the attribution values for each input feature.

Within this single-site retrospective study (Figure 1), clinical data and preoperative brain MRI images from 209 lesion-based sessions involving 130 patients who received therapy for brain metastasis at Wake Forest Baptist Comprehensive Cancer Center from 2011 to 2018 were gathered and analyzed. Since each session represents a separate radiotherapy treatment for an individual metastatic focus with heterogeneous outcomes, the instances were randomly divided into the training cohort (*n* = 125) and the validation cohort (*n* = 84).

This study utilized structural MRI images acquired with patients in a stereotactic head frame acquired on a 3.0T General Electric SIGNA Excite (GE Healthcare, Milwaukee, WI, USA). The dataset exclusively included T1 CE (contrast-enhanced) images.

Clinical information about oncology and radiotherapy treatment regimens [23,24,25,26,27] was collected, including the primary tumor site, histology, timing of immunotherapy, intact metastasis or post-operative resection cavity (the surgical cavity left), immune checkpoint inhibitor (ICI) use, prescription dose (the amount of radiation prescribed to the boundary of the target lesion), prescription isodose line (the percentage of the maximum dose used to define the treatment boundary), metastasis volume, the volume of the 12 Gy isodose line (v12), laterality, and location. The timing of immunotherapy is considered concurrent if SRS was received within 30 days before or after an ICI administration. Metastasis volume refers to the volume of the intact tumor for unresected lesions or the post-operative resection cavity for resected cases based on the MRI planning.

### 2.2. Radiosurgery Technique

Patients were treated on a Leksell Gamma Knife (Elekta AB, Stockholm, Sweden), and treatment planning was performed on the Leksell GammaPlan treatment planning system. On the day of treatment, a high-resolution, thin-slice, contrast-enhanced magnetic resonance imaging (MRI) scan was performed on a 3 T unit (GE Healthcare, Milwaukee, WI, USA). The median prescription dose to the metastasis margin was 20 Gy (interquartile range: 20–22 Gy). The dose prescription was based on the guidelines published by Shaw et al. [28]. All lesions were treated to the gross metastasis volume only with no additional clinical target volume or planning target volume.

### 2.3. Response Assessment and Necrosis Definition

Patients were followed up with an MRI of the brain approximately 6 to 8 weeks after SRS and then every 3 months for the first 2 years after SRS. After 2 years, imaging was generally performed less frequently. Radiation necrosis was defined based on the pathologic findings, if available, or the imaging features of MRI, such as increased T1 contrast enhancement, non-progression of the lesion over 4 months, and the absence of perfusion [29]. All diagnoses were confirmed retrospectively by the same experienced neuroradiological team.

### 2.4. Image Processing and Radiomic Feature Extraction

All brain MRI images were segmented to enhance the metastasis region. Initially, MRI images underwent image transformations and re-slicing using FSL (FMRIB Software Library) 6.0 scripts to ensure the images aligned correctly and consistently across all planes, maintaining uniform voxel dimensions. Subsequently, we utilized ITK-SNAP 4.2.0 software to place a bounding box around the suspected metastasis area automatically. Then, our trained research personnel manually traced the boundaries of metastases slice-by-slice [30]. These masks served as critical inputs to isolate and characterize the respective phenotypes during feature extraction.

Radiomic features were extracted from MRI images using a pipeline consisting of three sequential preprocessing steps: (1) skull-stripping using the Brain Extraction Tool (BET) and FreeSurfer to remove non-brain tissues from the MRI images [31]; (2) bias field correction using the N4ITKBiasFieldCorrection routine, implemented via nipype [32], to correct for intensity non-uniformity across the MRI images; (3) image intensity normalization using an algorithm to standardize the intensity scales across MRI images of the same contrast [33]. Subsequently, 386 initial radiomic features were extracted using the PyRadiomics [33] package on each derived image. The image processing and feature extraction process followed the Image Biomarker Standardization Initiative (IBSI) guidelines, with the IBSI reporting checklist provided in Supplementary Table S1 [34,35,36].

### 2.5. Feature Dimensionality Reduction

A dataset of 209 instances with 386 radiomic features and 9 clinical factors can lead to overfitting. To mitigate feature redundancy, we employed the L2 regularization technique with correlation analysis on the training cohort to reduce radiomics dimensionality [37]. Ridge regression (L2 regularization) was applied to estimate coefficients for each radiomic feature to the target variable. The coefficient values ranged from −0.2 to 0.2, with larger absolute values indicating a more substantial predictive impact. Features with absolute coefficient values were excluded. Then, we performed correlation analysis to identify correlations among the remaining features. Features with a correlation coefficient exceeding 0.8 were considered to exhibit strong linear relationships, suggesting potential redundancy. In cases of high correlation, the features with the smaller absolute coefficient value were removed.

Ultimately, this process selected 11 radiomic features from 386 initial ones, and their correlation coefficients are shown in Supplementary Figure S1. These features, with clinical factors, formed the predictive variables, which were used in subsequent modeling to predict radiation necrosis.

### 2.6. Model Development and Validation

This study employed an ensemble strategy to predict radiation necrosis. Specifically, the model utilized gradient boosting, random forest, decision tree, and support vector machine (SVM) as base regressors. A soft-voting regressor was then fitted with these four base regressors, aggregating their predictions to generate the final output. Additional models were used for performance comparison, including logistic regression, multi-layer perceptron classifier (MLP), k-nearest neighbors (KNNs), and Gaussian Naive Bayes (GaussianNB), which were employed using the scikit-learn library [38]. A stacking regressor strategy with the same basic models was employed as an alternative ensemble approach to compare. The synthetic minority over-sampling technique (SMOTE) from the imbalanced-learn library [39] was applied to the training cohort with a sampling strategy of 0.5, a random state of 42, and a k_neighbors of 5 to enhance the learning by generating synthetic minority samples. The soft-voting ensemble strategy and comparative models were evaluated using metrics, including the area under the curve (AUC), sensitivity, specificity, negative predictive value (NPV), and positive predictive value (PPV) [40]. Missing potential cases of radiation necrosis could have serious clinical consequences, so we prioritized sensitivity when selecting the threshold for all models. The clinical usefulness of the model was evaluated using decision curve analysis (DCA) [41]. All models were trained, validated, and hyperparameter-tuned using Python 3.9 on macOS 14.6.1, with scikit-learn 1.0.2 as the primary machine learning framework. The parameters for each regressor are provided in Supplementary Table S1, and the code is available on GitHub https://github.com/AI4Onc/mri-radiomics-ensemble-rn-bm-prediction (accessed on 18 March 2025).

### 2.7. Model Interpretation

Given the complexity of the predictive model with 20 predictors, interpreting its prediction is challenging. To address the black-box prediction challenge, we employed SHAP [18,19] as an explanatory model to analyze outputs from the ensemble model, providing insights into the model’s decision-making process, highlighting how each feature influences the likelihood of necrosis and offering a clearer understanding of the model’s prediction dynamics. This approach calculated SHAP values to measure the impact of each input feature on the predicted outcome. These values were used to rank features and visually represent key associations, where higher values indicate greater predictor significance and a stronger influence on the prediction of post-radiation necrosis occurrence. In addition, we utilized LIME to generate interpretable explanations for predictions as a supplement [20,21,22].

### 2.8. Statistical Analysis

The Mann–Whitney U test [42] was used to compare continuous variables, and the chi-squared test [43] was used to compare categorical variables, aiming to identify significant differences in clinical factors between groups. Both univariate and multivariate logistic regression analyses were conducted to evaluate the association between the model’s predicted outcome, clinical factors, and the occurrence of radiation necrosis [44,45]. All statistical analyses were conducted using a two-sided approach, considering a *p*-value smaller than 0.05 indicative of statistical significance.

## 3. Results

### 3.1. Clinical Characteristics

Comprehensive clinical factors regarding population, oncology information, and radiotherapy characteristics can be retrieved from Table 1. Out of the participants, 10 (8%) sessions in the training cohort and 5 (6%) in the validation cohort were classified as necrosis (Table 1). No significant differences were observed in the baseline characteristics between the necrosis and non-necrosis groups, except for patients’ age in the training cohort and metastasis volume and the volume of the 12 Gy isodose line in the validation cohort, which were statistically significant (*p* < 0.05).

### 3.2. Model Evaluation

**Figure 2:**
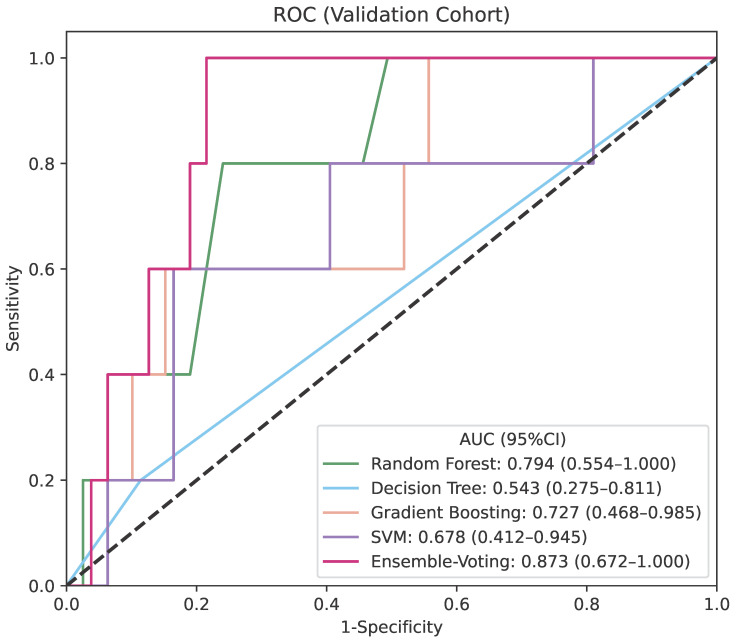
ROC (Receiver Operating Characteristic) curve plot displaying the prediction performance of the soft-voting ensemble model and individual prediction models in the validation cohort.

The ensemble model, combining predictions from gradient boosting, decision tree, random forest, and SVM using a soft-voting regressor, demonstrated predictive solid performance in the validation cohort with the highest AUC of 0.873 with a 95% confidence interval (CI) of 0.672–1.000 (Figure 2), indicating high discriminatory power in identifying patients at risk for radiation necrosis. While the ensemble model exhibited a training AUC comparable to that of individual base regressors (Supplemental Figure S2), it outperformed them in the validation cohort.

To ensure at-risk patients are accurately identified and receive appropriate preventive care or closer monitoring, we chose a threshold of 0.165, which maximizes the model’s sensitivity. The model achieved high sensitivity, NPV, and PPV, effectively identifying necrosis cases with minimal false negatives. Its specificity was 0.785 (0.694–0.875), suggesting a solid ability to correctly identify non-necrosis instances. Compared to individual models, the ensemble one consistently outperformed with superior overall metrics (Table 2). Specifically, gradient boosting achieved an AUC of 0.727 (0.468–0.985) and sensitivity of 0.600 (0.171–1.000); the random forest model reached an AUC of 0.794 (0.554–1.000) and sensitivity of 0.800 (0.449–1.000); the SVM model achieved an AUC of 0.678 (0.412–0.945) and sensitivity of 0.600 (0.171–1.000); while the decision tree model had the weakest performance with an AUC of 0.543 (0.275–0.811) and a sensitivity of 0.200 (0.000–0.551) (Table 2). These results indicate that our model was more effective at balancing sensitivity and specificity, making it more robust and reliable.

Additionally, a comparison was conducted between this soft-voting ensemble model and the ensemble model using staking regressor, as well as other commonly used models like logistic regression, MLP, KNNs, and GaussianNB. The stacking regressor also achieved good sensitivity and NPV but had a lower AUC of 0.828 (0.602–1.000) and specificity of 0.684 (0.581–0.786), as shown in Table 2. Its PPV is also lower than the soft-voting model and lower than the PPV of gradient boosting, random forest, and decision tree, reflecting a lower capacity to confirm positive cases. The performance of traditional models was notably lower than that of ensemble models. Logistic regression had an AUC of 0.466 (0.209–0.722). Similarly, MLP classification and KNNs demonstrated an AUC of 0.572 (0.302–0.842) and 0.627 (0.356–0.897), respectively. GaussianNB had the lowest AUC at 0.537 (0.270–0.804).

Combining outputs from multiple models into a single predictive model proved effective, minimizing false negatives while maintaining a solid balance between sensitivity and specificity. The ensemble model provided superior predictive accuracy compared to individual models and demonstrated robust performance in predicting radiation necrosis (Table 2).

**Figure 3:**
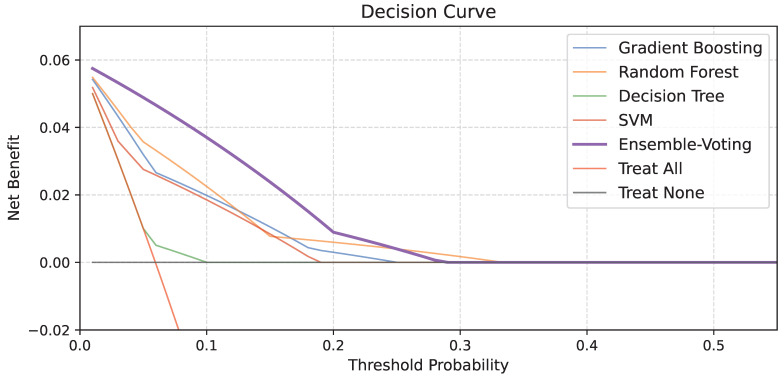
Decision curve analysis results comparing the proposed ensemble model with individual baseline classifiers.

In addition to traditional performance metrics, we evaluated the clinical usefulness of the proposed ensemble model and individual baseline classifiers using decision curve analysis (DCA) in Figure 3. The soft-voting ensemble model achieved a higher net benefit than the individual models across most of the clinically relevant threshold range and yielded the largest cumulative area above the “Treat None” line. These results suggest that the ensemble approach offers superior clinical utility compared to single-model baselines, particularly in the context of low disease prevalence.

### 3.3. Model Association with Radiation Necrosis

**Figure 4:**
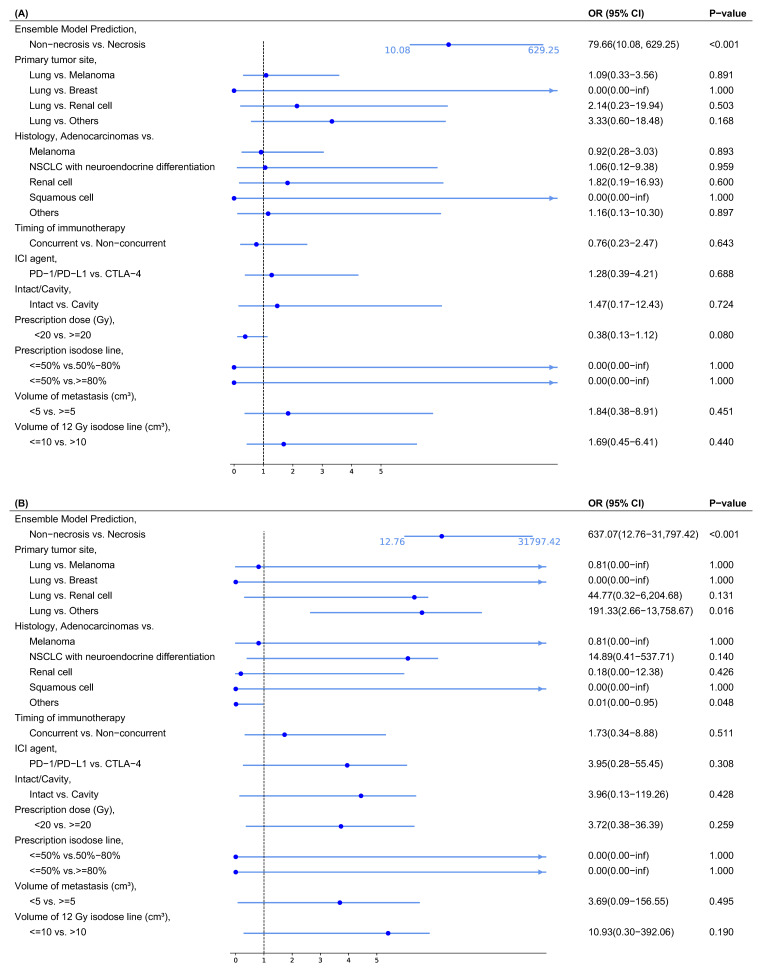
Forest plots for the univariate and multivariate logistic regression analyses results, including odds ratios (ORs) and *p*-values. (**A**) Univariate logistic regression analysis. (**B**) Multivariate logistic regression analysis.

The univariate logistic regression analysis on the entire dataset indicates that only the ensemble model prediction was significantly associated with radiation necrosis, demonstrating an odds ratio (OR) with a 95% CI of 79.66 (10.08–629.25) and a *p*-value < 0.001 (Figure 4A). This result suggests that the ensemble model serves as a strong and reliable predictor of radiation necrosis.

The multivariate logistic regression analysis on the entire dataset further confirmed that the ensemble model is the most significant predictor of radiation necrosis, with an OR of 637.07 (12.76–31,797.42) and a *p*-value < 0.001 (Figure 4B). This finding underscores the robustness of the ensemble model as a predictive tool, maintaining its significance even after adjusting for potential confounders. While a few factors were significant and demonstrated specific trends, most variables remained non-significant. Patients with primaries except for melanoma, lung, breast, and renal cell were more likely to develop necrosis compared to patients with lung as the primary metastasis, with an OR of 191.33 (2.66–13,758.67) and *p*-value of 0.016. Histology types, except for melanoma, non-small cell lung cancer (NSCLC) with neuroendocrine differentiation, squamous cell, and renal cell, showed a decreased likelihood of necrosis compared to adenocarcinomas, with an OR of 0.01 (0.00–0.95) and *p*-value of 0.048. Some non-significant features demonstrated trends, indicating potential predictive trends. The renal cell primary site was more likely to have necrosis than the lung, with an OR of 44.77 (0.32–6,204.68). Histology of NSCLC with neuroendocrine differentiation had a higher probability of necrosis compared to adenocarcinomas, with an OR of 14.89 (0.41–537.71). Non-concurrent treatment had a lower likelihood of necrosis than concurrent one, with an OR of 1.73 (0.34–8.88). Using CTLA-4 was associated with a higher risk, as evidenced by an OR of 3.95 (0.28–55.45). Post-operative cavities were more likely to develop radiation necrosis, with an OR of 3.96 (0.13–119.26). The OR value of metastasis volume (<5 vs. ≥5 cm^3^) was 3.69 (0.09–156.55), and the value of v12 (≤10 vs. >10 cm^3^) was 10.93 (0.30–392.06).

The results of univariate and multivariate logistic regression analyses on the validation cohort (Supplementary Figure S4) indicate a trend suggesting that model prediction is more strongly associated with radiation necrosis than other clinical factors, despite limited statistical power due to the small sample size. In the multivariate logistic regression analysis, all predictors had *p*-values greater than 0.95, except for model prediction and timing of immunotherapy. The model prediction had an OR greater than 1, indicating it was the strongest positive predictor within the validation cohort. Although the results did not reach statistical significance due to the limited sample size, the model prediction still demonstrated a clear trend as the strongly predictive factor with radiation necrosis, further supporting its potential predictive relevance.

Overall, the logistic regression analyses strongly support the ensemble model’s predictive capacity for radiation necrosis. The ensemble model’s consistent statistical significance across analyses demonstrates its robustness as an independent predictor.

### 3.4. Feature Importance and Clinical Relevance

Among the 11 selected radiomic features (Supplementary Table S2), the majority are texture-based features derived from GLCM, GLDM, and GLSZM matrices, highlighting the importance of spatial heterogeneity and microstructural irregularity in distinguishing radiation necrosis. There are eight second-order texture features (Imc2, Idmn, ZonePercentage, DependenceVariance, DependenceNonUniformityNormalized, SmallDependenceLowGrayLevelEmphasis, and SmallAreaLowGrayLevelEmphasis), which capture patterns of voxel intensity relationships and local tissue disorganization. Most features are extracted after applying the Laplacian of Gaussian filters at varying spatial scales (log-sigma), enabling the capture of textural patterns at both fine and coarse resolutions. The dominance of texture-related features is consistent with the clinical observation that radiation necrosis exhibits greater local heterogeneity and irregular intensity distributions on CT imaging.

As shown in Supplementary Figure S3A,B, metastasis volume emerged as the most significant factor, while log-sigma-1-mm_glcd_ldmn ranked second. These results suggest that higher values of these features correspond to a greater likelihood of radiation necrosis. We further used SHAP for both global and local interpretations. In the force plot (Supplementary Figure S3C) and decision plot (Supplementary Figure S3D), the prediction process is visualized, showing that, in instance 1, the model’s baseline probability of necrosis is 0.29, increasing to 0.38 based on factors like the volume of metastasis and log-sigma-1-mm_glcd_ldmn.

Additionally, LIME was used as a complementary method, focusing on the model’s local prediction dynamics. The most influential features are original_gldm_DependenceNonUniformityNormalized, metastasis volume, and log-sigma-1-mm-3D_firstorder_InterquartileRange (Supplementary Figure S3E), which contribute significantly to reducing the likelihood of necrosis. Conversely, log-sigma-3-mm-3D_glcm_Idmn showed a positive association.

Both analyses identify metastasis volume as the most significant predictor, highlighting the critical role of metastasis size in influencing radiation necrosis. The radiomic features used as predictors include GLCM-based texture features, GLDM features, skewness, dependence variance, and Imc2, along with features characterizing fine structural patterns, tissue density, and local textural variations. Among them, log-sigma-1-mm-3D_glcm_Idmn, log-sigma-3-mm-3D_glcm_Idmn, and original_gldm_DependenceNonUniformityNormalized were identified as the top three most influential radiomics by both analyses, emphasizing that texture homogeneity and the non-uniformity of gray-level dependencies play a crucial role in prediction. The results align with the clinical understanding that these factors are key contributors to radiation-induced damage and necrosis formation.

## 4. Discussion

In this study, we developed an ensemble model incorporating radiomic features and clinical factors to predict radiation necrosis for patients with brain metastasis taking SRS-ICI therapy. The crucial task of predicting radiation necrosis can be effectively accomplished by utilizing this model. As evidenced by a favorable AUC, along with high sensitivity, specificity, PPV, and NPV, the ensemble model demonstrates excellent performance in accurately distinguishing the sessions that are more likely to develop radiation necrosis. Furthermore, we employed SHAP and LIME analyses to interpret the model’s predictions and demonstrate that its predictions are clinically meaningful and rational. Our study offers a dependable and reproducible tool for the pretreatment prediction of radiation necrosis, thereby facilitating the clinical implementation of computer-assisted case-customized management of patients diagnosed with brain metastasis. By accurately stratifying treatment sessions based on their risk of necrosis before initiating radiotherapy, the model aids in customizing treatment decisions, facilitates proactive monitoring for earlier prognosis, and helps reduce adverse outcomes.

In the context of brain metastasis, radiation necrosis is a significant treatment-related complication that can severely affect patient quality of life and clinical outcomes [3]. Radiomics, as a promising tool in brain metastasis, is primarily used to predict radiation response or monitor metastasis [6,7,8,9,10], which offers insights into tumor biology, microenvironment, and treatment effects that may not be visible through traditional imaging evaluation. Current radiomics-based applications primarily focus on predicting treatment response [11] or identifying brain metastasis recurrence from radiation necrosis [12,13]. However, predicting radiation necrosis has yet to be explored. Accurate prediction of necrosis is critical for risk stratification, treatment planning, and patient counseling, offering a proactive approach to managing potential complications.

The proposed MRI-based radiomics ensemble model fills this gap by leveraging advanced machine learning techniques to estimate the necrosis of patients who received SRS-ICI treatment. This predictive capacity is clinically meaningful as it allows for identifying the session at high risk for necrosis early in the treatment cycle. Such predictions enable clinicians to modify treatment strategies, optimize dose delivery, or incorporate preventive measures to mitigate necrosis risk, thus improving patient management. Furthermore, it can help to make an earlier prognosis, potentially reducing adverse outcomes, improving survival rates, and enhancing the overall quality of life for patients. By addressing the predictive need for radiation necrosis, this model represents a significant step forward in computer-assisted treatment management for patients diagnosed with brain metastasis.

Our study has several limitations. These include bias due to the retrospective data sources. It requires further validation on larger independent prospective cohorts comprising homogeneous patient populations and image modalities. Second, this model demonstrates potential in predicting radiation necrosis in radiotherapy sessions for brain metastasis. However, the current dataset is limited to SRS-ICI treatment. To enhance its generalizability for clinical use, the model requires further training and validation on instances involving SRS alone and combined with other intracranially active systemic therapies. Third, for the OR results, the significant findings related to the primary tumor site and histology may have been influenced by the bias from different category distributions, which should be further validated using larger and more balanced datasets.

## 5. Conclusions

We have developed and validated an MRI-based radiomics ensemble model to predict radiation necrosis in treatment sessions involving combined SRS-ICI therapy for brain metastasis. Through validation, our model demonstrates superior predictive value compared to traditional machine learning algorithms. Further research and validation are still essential to confirm these findings and assess the clinical applicability of the model. Large-scale prospective trials will be crucial to refine our results and determine the model’s effectiveness in guiding case-customized radiotherapy or alternative treatment strategies for patients with brain metastases.

## Figures and Tables

**Table 1:** Clinical characteristics.

		Training Cohort (n = 125)			Validation Cohort (n = 84)		
		Necrosis (n = 10)	Non-Necrosis (n = 115)	*p*-Value	Necrosis (n = 5)	Non-Necrosis (n = 79)	*p*-Value
Gender				0.465			0.739
	Female	6 (60)	49 (43)		3 (60)	33 (42)	
	Male	4 (40)	66 (57)		2 (40)	46 (58)	
Age				0.041			0.449
	<=60	7 (70)	43 (37)		2 (40)	27 (34)	
	>60	3 (30)	72 (63)		3 (60)	52 (66)	
Death				0.737			0.572
	Dead	5 (50)	70 (61)		2 (40)	50 (63)	
	Not Dead	5 (50)	45 (39)		3 (60)	29 (37)	
Primary tumor site				0.418			0.390
	Lung	5 (50)	61 (53)		2 (40)	44 (56)	
	Melanoma	3 (30)	40 (35)		2 (40)	29 (37)	
	Breast	0 (0)	4 (3)		0 (0)	0 (0)	
	Renal cell	0 (0)	4 (3)		1 (20)	3 (4)	
	Other	2 (20)	6 (5)		0 (0)	3 (4)	
Histology				0.820			0.357
	Adenocarcinomas	6 (60)	53 (46)		1 (20)	36 (46)	
	NSCLC with neuroendocrine differentiation	0 (0)	9 (8)		1 (20)	3 (4)	
	Melanoma	3 (30)	40 (35)		2 (40)	29 (37)	
	Renal cell carcinoma	0 (0)	3 (3)		1 (20)	4 (5)	
	Squamous cell carcinoma	0 (0)	4 (3)		0 (0)	2 (3)	
	Other	1 (10)	6 (5)		0 (0)	5 (6)	
Laterality				0.766			0.008
	Left	6 (60)	61 (53)		0 (0)	35 (44)	
	Right	4 (40)	49 (43)		4 (80)	43 (54)	
	Midline	0 (0)	5 (4)		1 (20)	1 (1)	
Location				0.984			0.095
	Frontal	5 (50)	45 (39)		0 (0)	28 (35)	
	Parietal	1 (10)	19 (17)		0 (0)	14 (18)	
	Cerebellar	1 (10)	13 (11)		1 (20)	12 (15)	
	Temporal	1 (10)	10 (9)		3 (60)	8 (10)	
	Occipital	1 (10)	11 (10)		1 (20)	11 (14)	
	Basal ganglia	0 (0)	3 (3)		0 (0)	2 (3)	
	Brainstem	0 (0)	6 (5)		0 (0)	1 (1)	
	Periventricular	0 (0)	2 (2)		0 (0)	3 (4)	
	Other	1 (10)	6 (5)		0 (0)	0 (0)	
Timing of immunotherapy				0.917			1.000
	Concurrent	8 (80)	84 (73)		3 (60)	47 (59)	
	Non-concurrent	2 (20)	31 (27)		2 (40)	32 (41)	
ICI agent				1.000			0.842
	PD−1/PD−L1	8 (80)	92 (80)		3 (60)	59 (75)	
	CTLA−4	2 (20)	23 (20)		2 (40)	20 (25)	
Intact/Cavity				1.000			0.571
	Intact	10 (100)	109 (95)		4 (80)	76 (96)	
	Cavity	0 (0)	6 (5)		1 (20)	3 (4)	
Prescription dose (Gy)				0.554			0.252
	<20	4 (40)	24 (21)		2 (40)	15 (19)	
	>=20	6 (60)	91 (79)		3 (60)	64 (81)	
Prescription isodose line				0.160			0.336
	<=50%	10 (100)	91 (79)		5 (100)	66 (84)	
	50–80%	0 (0)	15 (13)		0 (0)	7 (8)	
	>=80%	0 (0)	9 (8)		0 (0)	6 (8)	
Volume of metastasis (cm^3^)				0.106			0.009
	<5	10 (100)	104 (90)		3 (60)	75 (95)	
	>=5	0 (0)	11 (10)		2 (40)	4 (5)	
V12 (cm^3^)				0.066			0.013
	<=10	9 (90)	97 (84)		3 (60)	72 (91)	
	>10	1 (10)	18 (16)		2 (40)	7 (9)	

Abbreviations: NSCLC = non-small cell lung cancer; ICI = immune checkpoint inhibitor; PD−1/PD−L1 = programmed cell death protein 1/programmed death-ligand 1; CTLA−4 = Cytotoxic T-Lymphocyte Antigen 4; V12 = volume of 12 Gy isodose line.

**Table 2:** Prediction performance of soft-voting ensemble model versus stacking ensemble model and individual prediction models in the validation cohort (95%).

	AUC	Sensitivity	Specificity	NPV	PPV
**Soft-voting Ensemble Model**	**0.873 (0.672−1.000)**	**1.000 (1.000−1.000)**	**0.785 (0.694−0.875)**	**1.000 (1.000−1.000)**	**0.227 (0.052−0.402)**
Stacking Ensemble Model	0.828 (0.602−1.000)	1.000 (1.000−1.000)	0.684 (0.581−0.786)	1.000 (1.000−1.000)	0.167 (0.033−0.300)
Gradient Boosting	0.727 (0.468−0.985)	0.600 (0.171−1.000)	0.848 (0.769−0.927)	0.971 (0.931−1.000)	0.200 (0.000−0.402)
Random Forest	0.794 (0.554−1.000)	0.800 (0.449−1.000)	0.759 (0.665−0.854)	0.984 (0.952−1.000)	0.174 (0.019−0.329)
Decision Tree	0.543 (0.275−0.811)	0.200 (0.000−0.551)	0.886 (0.816−0.956)	0.946 (0.894−0.997)	0.100 (0.000−0.286)
Support Vector Machine	0.678 (0.412−0.945)	0.600 (0.171−1.000)	0.835 (0.754−0.917)	0.971 (0.930−1.000)	0.188 (0.000−0.379)
Logistic Regression	0.466 (0.209−0.722)	0.400 (0.000−0.829)	0.823 (0.739−0.907)	0.956 (0.907−1.000)	0.125 (0.000−0.287)
Multi-Layer Perceptron Classifier	0.572 (0.302−0.842)	0.600 (0.171−1.000)	0.696 (0.595−0.798)	0.965 (0.917−1.000)	0.111 (0.000−0.230)
K-Nearest Neighbors	0.627 (0.356−0.897)	0.600 (0.171−1.000)	0.620 (0.513−0.727)	0.961 (0.908−1.000)	0.091 (0.000−0.189)
Gaussian Naive Bayes	0.537 (0.270−0.804)	0.400 (0.000−0.829)	0.810 (0.724−0.897)	0.955 (0.906−1.000)	0.118 (0.000−0.271)

Abbreviations: AUC = Area Under the Curve; NPV = Negative Predictive Value; PPV = Positive Predictive Value.

## Data Availability

The data supporting this study are not publicly available due to patient privacy restrictions. However, data access requests may be considered by the corresponding author upon reasonable request and with appropriate institutional approval.

## Supplementary Materials

The following supporting information can be downloaded at: https://www.mdpi.com/article/10.3390/cancers17121974/s1, Figure S1: Radiomics correlation heatmap. Figure S2: ROC (Receiver Operating Characteristic) curve plot. Figure S3: (A) Beeswarm summary plot of feature importance from SHapley Additive exPlanations (SHAP) analysis. (B) Feature importance plot. (C) SHAP force plots. (D) SHAP decision plot. (E) Local interpretable model-agnostic explanations (LIME) analysis plot. Figure S4: Forest plots for univariate and multivariate logistic regression analyses results on the validation cohort exclusively, including odds ratios (OR) and p-values. (A) Univariate logistic regression analysis. (B) Multivariate logistic regression analysis. Table S1: Image Biomarker Standardisation Initiative (IBSI) reporting Checklist. Table S2: Selected Radiomics Summary.
