# Supplementary material for: MRI-Based Radiomics Ensemble Model for Predicting Radiation Necrosis in Brain Metastasis Patients Treated with Stereotactic Radiosurgery and Immunotherapy
Source: Cancers (Basel). 2025 Jun 13;17(12):1974. doi: 10.3390/cancers17121974 (PMC12191015; doi:10.3390/cancers17121974)
Supplement: Supplementary file 1 [file cancers-17-01974-s001.zip › Supplemental Table S1.pdf]

Supplementary Table S1: Image Biomarker Standardization Initiative (IBSI) Checklist

Topic	Item	Description
**Patient**		
Region of interest	1	Brain
Patient preparation	2a	Patients are not required to fast prior to MRI imaging.<br>Removal of metallic objects is mandatory to avoid MRI artifacts.<br>Patients are instructed to remain as still as possible during the scan to minimize motion artifacts.
	2b	If contrast-enhanced MRI is performed, a Gadolinium-based contrast agent is administered intravenously prior to image acquisition. The standard dosage is 0.1–0.2 mmol/kg of body weight.<br>For claustrophobic patients, a mild sedative may be given.<br>Muscle relaxants are not routinely used unless required for patients with conditions that cause involuntary movements.
	2c	Earplugs or noise-canceling headphones are provided to reduce discomfort from MRI scanner noise.<br>Cushions and head restraints are used to minimize involuntary head movements.<br>Temperature control inside the scanner is maintained to ensure comfort.<br>Verbal communication via an intercom system is maintained to monitor patient well-being during the scan.
Contrast agent	4a	Gadolinium-based contrast
	4b	The contrast agent is administered intravenously (IV) using a power injector or manual bolus injection.
	4c	The standard dosage follows body weight-based dosing:<br>0.1–0.2 mmol/kg of body weight.
	4d	The typical uptake time is 5–10 minutes post-injection before acquiring post-contrast T1-weighted imaging.
	4e	Renal function is assessed before contrast administration using serum creatinine or eGFR measurements to minimize nephrogenic systemic fibrosis (NSF) risk.<br>Hydration status is considered, particularly in patients with renal impairment, to reduce contrast agent retention.<br>Patients with prior allergic reactions to Gadolinium are pre-medicated with antihistamines or corticosteroids if necessary.
Comorbidities	5	Patients with renal impairment (eGFR < 30 mL/min/1.73m^2) require careful contrast agent dose adjustment or alternative imaging.<br>Diabetic patients taking metformin may require temporary discontinuation of the drug to avoid contrast-induced lactic acidosis.<br>Patients with motion disorders may be given mild sedatives or instructed to keep still to prevent motion artifacts.<br>Patients with metallic implants undergo safety screening before MRI to ensure compatibility.<br>History of prior radiation therapy may alter tissue contrast and signal intensity in imaging.
**Acquisition**		

Acquisition protocol	6	A standardized imaging protocol was used for all patients to ensure consistency and reproducibility across scans.<br>The protocol description follows established guidelines for brain MRI tumor imaging and can be found in institutional imaging protocols or referenced literature.
Scanner type	7	GE HealthCare - Signa Excite 3.0T
Imaging modality	8	Magnetic Resonance Imaging (MRI)
Scanner calibration	9	Routine calibration was performed following GE Healthcare’s manufacturer guidelines to ensure imaging consistency.
Acquisition type	10a	Static
Scan duration	11	T1-weighted (pre-contrast): ~5 minutes
Patient instructions	12	Patients were instructed to remain still throughout the scan to minimize motion artifacts.<br>Verbal instructions via an intercom system were provided before and during scanning.
Anatomical motion correction	13	Motion correction strategies were implemented to minimize the impact of involuntary patient movement:<br>Head restraints and foam padding were used to reduce head motion inside the MRI head coil. Physiological motion correction was not required, as brain MRI is less affected by respiratory or cardiac motion. Retrospective motion correction software was applied if necessary.<br>If excessive motion artifacts were detected, sequences were reacquired to ensure image quality.
Magnetic field strength	19	The MRI scans were acquired at a nominal field strength of 3.0T, using the GE Healthcare - Signa Excite 3.0T scanner.
RF coil	20	The RF coil used for image acquisition was a dedicated head coil.
Acquisition type	21	The MRI acquisition type was 3D volumetric imaging, allowing for isotropic voxel resolution and better tumor segmentation.
Scanning sequence	22a	T1-weighted, pre-contrast
	22b	T1-weighted imaging used a Spin Echo (SE) variant.
	22c	Flow compensation was enabled to reduce motion artifacts from cerebrospinal fluid (CSF) pulsations.
	22d	Repetition Time: 800 ms
	22e	Echo time: 19 ms
	22f	No inversion time, 0
	22g	Flip Angle: 90
	22h	The standard Cartesian (linear) k-space trajectory.
	22i	Each k-space point was sampled once for standard sequences.
	22j	For standard sequences, each RF excitation pulse covered one line of k-space.
**Reconstruction**		
Resolution	23a	Field of View (FOV): ~250 × 250 mm^2<br>Matrix Size: 512 × 512
	23b	1 × 1 × 1 mm^3 resolution
MRI reconstruction method	27a	Standard Fourier Transform-based reconstruction was used to convert raw k-space data into spatial domain images.
	27b	Parallel imaging reconstruction (ASSET for GE scanners) was applied to reduce aliasing artifacts from undersampling.

**Image processing - post-acquisition processing**		
Anti-aliasing	33	Bicubic interpolation was used to prevent aliasing artifacts during image down-sampling.<br>In cases requiring voxel resampling (to standardize voxel dimensions to 1 × 1 × 1 mm³), B-spline interpolation was applied to ensure smooth transitions between resampled voxels.
Noise suppression	34	Non-local means (NLM) filtering to reduce random noise while preserving structural details.<br>Wavelet denoising was applied for fine-scale noise removal, especially for MRI images with low SNR.<br>Gaussian filtering was used in specific cases to reduce speckle noise.
Skull stripping	36	BET (Brain Extraction Tool) from FSL
Non-uniformity correction	37	N4ITK bias field correction was applied to compensate for intensity inhomogeneity across MRI scans.<br>The correction parameters included: Shrink factor = 3–4 (to speed up processing), smoothing kernel size = 50 mm to remove low-frequency bias, convergence threshold = 0.0001 for stability.
Intensity normalization	38	Z-score normalization (mean = 0, standard deviation = 1) was applied within each patient’s image to standardize intensity distribution.
Augmentation/perturbation methods	39	Histogram equalization and contrast stretching to account for intensity variations across different MRI scanners.
Other post-acquisition processing methods	40	Voxel-wise standardization was applied for feature stability.<br>Edge-preserving filters were used to enhance structural boundaries without excessive smoothing.<br>ROI mask refinement was performed using morphological operations (e.g., dilation and erosion) to remove small segmentation artifacts.

**Segmentation**		
Segmentation method	41a	Brain Tumor Segmentation (BraTS) Challenge criteria.
	41b	Manual segmentation by experienced radiologists.
	41c	Gross Tumor Volume (GTV) was segmented, including all visible tumor regions but excluding peritumoral edema unless explicitly required.<br>Necrotic/cystic tumor areas were included within the segmentation unless they were clearly non-tumoral.<br>Lymph nodes were not included in the segmentation unless explicitly analyzed as part of tumor spread.
	41d	Manual segmentation was performed by two board-certified neuroradiologists with at least 5 years of experience in brain MRI interpretation.
	41e	Semi-Automatic Segmentation:<br>Region-growing and threshold-based methods were used to generate an initial segmentation.<br>Morphological operations were applied to refine segmentation boundaries.

Conversion to mask	42	The segmented regions were converted into voxel-based binary masks for radiomics analysis.<br>Method for mask conversion:<br>Polygonal segmentations were rasterized onto a 3D voxel grid to match the original MRI spatial resolution.<br>Interpolation methods (e.g., nearest-neighbor or trilinear interpolation) were used if segmentation was performed at a different resolution.<br>The final binary mask had a voxel value of 1 for tumor regions and 0 for the background.
**Image processing - image interpolation**		
Interpolation method	43a	B-Spline interpolation was used for most image resampling to ensure smooth intensity transitions.<br>Nearest-neighbor interpolation was used for segmentation masks to preserve binary labels without introducing artifacts.
	43b	The interpolation grid was aligned by the center of the original image to maintain anatomical consistency across resampled images.<br>This ensures that resampled images retain spatial alignment with the original dataset.
	43c	The grid dimensions were rounded to the nearest integer voxel size to maintain uniformity in resampled images.<br>If non-integer dimensions resulted from resampling, the size was adjusted to the closest integer value to maintain standard voxel spacing.
	43d	Zero-padding was applied beyond the original image boundaries to avoid introducing artificial values.<br>In cases where missing data occurred at the edges, mirror padding or nearest-neighbor extrapolation was used to maintain continuity.
Voxel dimensions	44	The interpolated voxel dimensions were standardized to $1 \times 1 \times 1$ mm³ for all MRI scans.
**Image processing - ROI interpolation**		
Interpolation method	46	Nearest-neighbor interpolation was used for ROI mask resampling to ensure that binary labels (0 for background, 1 for tumor) were preserved without introducing intermediate values.
Partially masked voxels	47	Binary thresholding was applied (≥ 0.5 thresholding) after nearest-neighbor interpolation to ensure that partially masked voxels were assigned to either the tumor or background category.<br>In cases where a voxel was partially included in the mask due to interpolation, it was assigned a label of 1 (tumor) if more than 50% of the voxel overlapped with the segmented region.<br>Morphological filtering (e.g., dilation/erosion) was used post-interpolation to refine mask boundaries.
**Image processing - discretisation**		
Discretisation method	49a	Fixed bin count discretization was used for radiomics feature extraction.
	49b	A fixed bin count of 25 was applied to maintain reproducibility.
	49c	The lowest intensity in the first bin was set to the minimum intensity of the tumor region in the resampled image.
**Image processing - image transformation**		

Image filters	50a	Laplacian of Gaussian (LoG) filtering was applied for multi-scale texture feature extraction.<br>Wavelet filtering was used to decompose images into frequency-based components.
	50b	Image transformations were applied after interpolation and intensity normalization to ensure that extracted features were spatially stable across patients.
	50c	LoG Sigma Values: [1.0, 2.0, 3.0], ensuring multi-scale texture analysis.<br>Wavelet decomposition levels: All high-pass and low-pass filter combinations were computed.
	50d	First-order statistical features (e.g., mean, entropy, skewness).<br>Texture features from GLCM, GLDM, GLSZM, and GLRLM were computed from the response maps of the filtered images.
IBSI compliance	51	The software used for image transformations and feature extraction (PyRadiomics) is compliant with the Image Biomarker Standardization Initiative (IBSI).
**Feature computation**		
Feature set	52	The following hand-crafted radiomics features were extracted, following IBSI (Image Biomarker Standardization Initiative) definitions:<br>1. First-Order Statistics (describing voxel intensity distributions):<br>Mean, Variance, Skewness, Kurtosis, Entropy, Energy, and Total Energy; 10th, 25th, 50th, 75th, and 90th percentile Robust Mean Absolute Deviation (rMAD)<br>2. Shape-Based Features (quantifying the tumor's 3D morphology):<br>Volume, Surface Area, Sphericity, Compactness, Elongation, Maximum 3D Diameter, Surface-to-Volume Ratio<br>3. Texture Features (matrix-based descriptors of tumor heterogeneity):<br>Gray-Level Co-occurrence Matrix (GLCM): Contrast, Correlation, Energy, Homogeneity, Entropy; Gray-Level Run Length Matrix (GLRLM): Short Run Emphasis, Long Run Emphasis, Run Percentage; Gray-Level Size Zone Matrix (GLSZM): Small Area Emphasis, Large Area High Gray Level Emphasis; Gray-Level Dependence Matrix (GLDM): Dependence Entropy, Dependence Variance; Neighborhood Gray-Tone Difference Matrix (NGTDM): Coarseness, Strength, Contrast<br>4. Filtered Features (extracting multi-scale spatial information):<br>Laplacian of Gaussian (LoG, σ = 1.0, 2.0, 3.0) and Wavelet decomposition (all LL, LH, HL, HH sub-bands)<br>Feature computation was performed using PyRadiomics, ensuring IBSI-compliance and feature standardization.
Texture parameters	53a	GLCM, GLRLM, GLSZM, GLDM, and NGTDM matrices were computed across all 13 directions in 3D space, then averaged (merged) across directions to generate a single set of texture features.
	53b	No weighting was applied to the co-occurrence matrices.
	53c	Symmetric co-occurrence matrices were computed, meaning each intensity pair (i, j) was counted both forward and backward.
	53d	Chebyshev distance = 1 was used to determine voxel intensity co-occurrence relationships.

	53e	Chebyshev distance = 1 was applied when defining zones in GLSZM/DZM.<br>Voxel connectivity = 26-connected neighborhood in 3D was used for zone grouping.
	53f	Manhattan distance was used to compute the proximity of zones to ROI boundaries.
	53g	Chebyshev distance = 1 was used for defining local neighborhoods in NGTDM and NGLDM.
	53h	Coarseness parameter = 0, ensuring that all intensity differences within the neighborhood contribute equally to the dependence matrix.
IBSI compliance	54	The PyRadiomics framework was used for feature extraction.<br>PyRadiomics has been validated against IBSI reference values, ensuring reproducibility and compliance with IBSI standards for radiomics features.
**Machine learning and radiomics analysis**		
Diagnostic and prognostic modelling	55	The radiomics model development followed the TRIPOD (Transparent Reporting of a Multivariable Prediction Model for Individual Prognosis or Diagnosis) guidelines for reporting predictive models.<br>The model was trained to differentiate between patients with and without radiation necrosis (RN) based on radiomics features.<br>Performance metrics included AUC-ROC, accuracy, sensitivity, specificity, F1-score, and precision-recall analysis.
Robustness	56	Two independent radiologists segmented the same images, and Dice similarity coefficients (DSC) were calculated.<br>Features with DSC < 0.80 were excluded from the final model to ensure reproducibility.
Comparison with known factors	57	The performance of radiomics-based models was compared to traditional clinical risk factors such as: tumor volume, prescription dose and V12 (12 Gy isodose volume), and use of immunotherapy (ICI agent presence)
Multicollinearity	58	L2-regression and correlation analysis to detect multicollinearity
Model availability	59	GitHub repository: https://github.com/AI4Onc/mri-radiomics-ensemble-rn-bm-prediction<br>The Gradient Boosting Classifier (GB) was set with a learning rate of 0.01, 200 estimators, and max depth of 5 to prevent overfitting while ensuring convergence.<br>The Random Forest Classifier (RF) used 35 estimators, balanced class weights, and max depth of 5 to improve handling of class imbalance.<br>The Decision Tree Classifier (DT) applied a class weight of {0:1, 1:3} and max depth of 6, ensuring a higher penalty for misclassification of the minority class.<br>The Support Vector Machine (SVM) was configured with class weights {0:1, 1:3}, probability estimation enabled, and 3000 max iterations for improved convergence.
Data availability	60	Data availability is governed by IRB (Institutional Review Board) and patient privacy regulations. It is available on request.
Software	61	Image Processing & Feature Extraction: PyRadiomics (v3.0.1)<br>Machine Learning and Model Training: Python 3.9; Scikit-learn (v1.0.2) – for traditional ML models (Random Forest, SVM, Gradient Boosting).
