# Supplementary material for: MRI-Based Radiomics Ensemble Model for Predicting Radiation Necrosis in Brain Metastasis Patients Treated with Stereotactic Radiosurgery and Immunotherapy
Source: Cancers (Basel). 2025 Jun 13;17(12):1974. doi: 10.3390/cancers17121974 (PMC12191015; doi:10.3390/cancers17121974)
Supplement: Supplementary file 1 [file cancers-17-01974-s001.zip › Supplemental Table S2.pdf]

Supplementary Table S2: Selected Radiomics Summary

Radiomic Feature	Image Characteristic	Biological Characteristic
log-sigma-2-mm-3D_glcm_Imc2	Texture complexity	Measures informational complexity. High values indicate increased heterogeneity and disordered tissue structure.
original_firstorder_Energy	Global intensity	Sum of squared voxel intensities that may be lower in necrotic regions with reduced signal enhancement.
log-sigma-2-mm-3D_gldm_SmallDependenceLowGrayLevelEmphasis	Low-intensity texture pattern	Highlights small, weakly enhancing structures, which is typical of necrotic granularity.
log-sigma-3-mm-3D_glszm_ZonePercentage	Structural fragmentation	Higher values indicate more fragmented, heterogeneous zones. Common in necrosis.
log-sigma-3-mm-3D_gldm_DependenceVariance	Texture irregularity	Captures variability in voxel dependence. Higher in disorganized, necrotic tissue.
log-sigma-3-mm-3D_glszm_SmallAreaLowGrayLevelEmphasis	Localized low-intensity zone	Indicates prevalence of small, dimly enhancing regions seen in necrotic lesions.
log-sigma-2-mm-3D_firstorder_Skewness	Intensity asymmetry	Measures asymmetry in voxel intensity distribution, showing deviation from normal tissue patterns.
log-sigma-1-mm-3D_firstorder_InterquartileRange	Intensity spread	Wider interquartile range suggests greater heterogeneity within the lesion.
original_gldm_DependenceNonUniformityNormalized	Texture heterogeneity	High values indicate uneven texture patterns. Common in necrosis.
log-sigma-3-mm-3D_glcm_Idmn	Texture homogeneity	Measures local homogeneity. Lower in highly irregular tissue.
log-sigma-1-mm-3D_glcm_Idmn	Texture homogeneity	Same as above at a finer scale, which reduced in necrotic regions.
