# Supplementary figures and images for: MRI-Based Radiomics Ensemble Model for Predicting Radiation Necrosis in Brain Metastasis Patients Treated with Stereotactic Radiosurgery and Immunotherapy
Source: Cancers (Basel). 2025 Jun 13;17(12):1974. doi: 10.3390/cancers17121974 (PMC12191015; doi:10.3390/cancers17121974)

Radiomic Features Correlation Heatmap

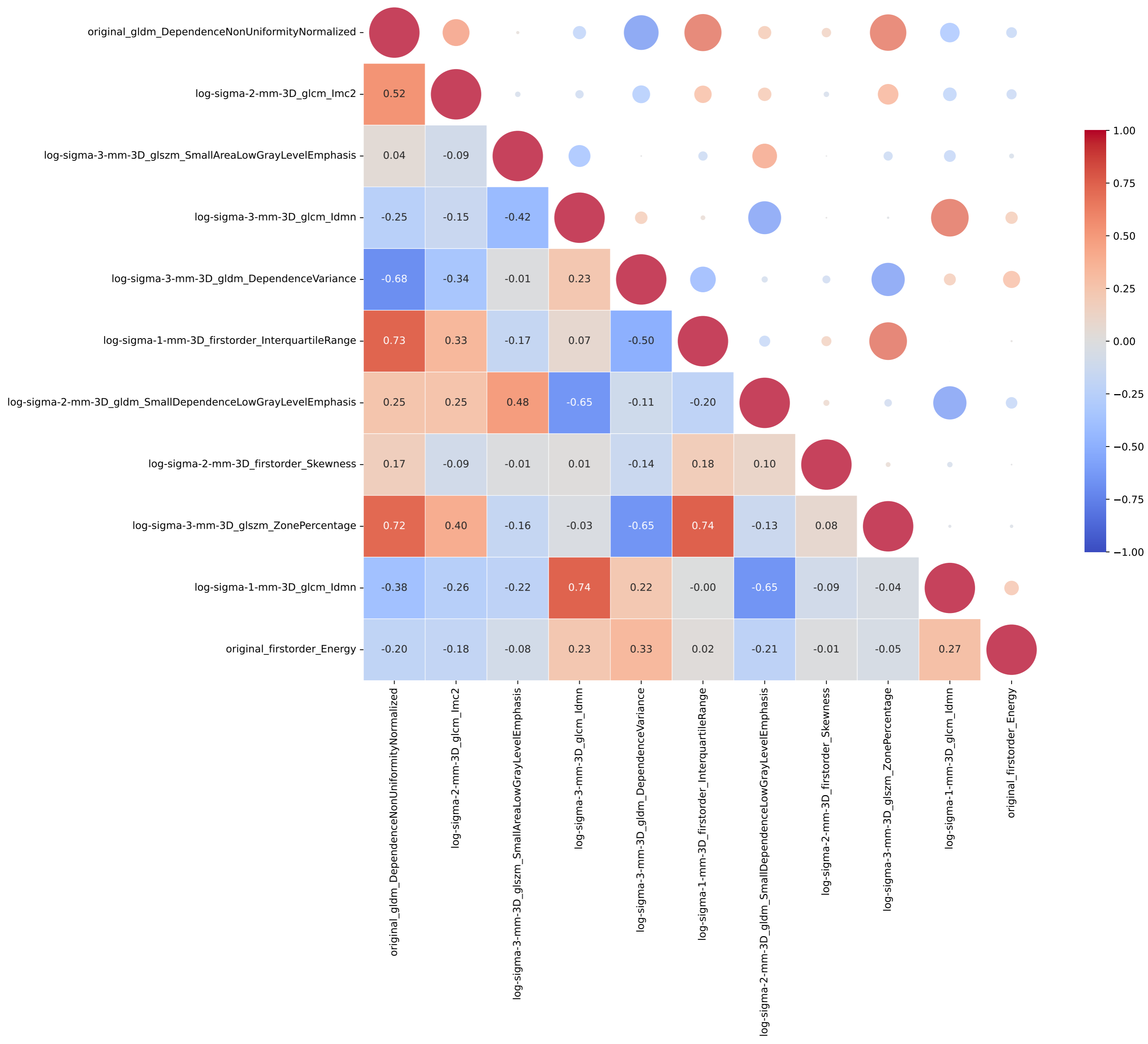

Supplement: Supplementary file 1 [file cancers-17-01974-s001.zip › Supplement Figure S1.pdf]

# ROC (Training Cohort)

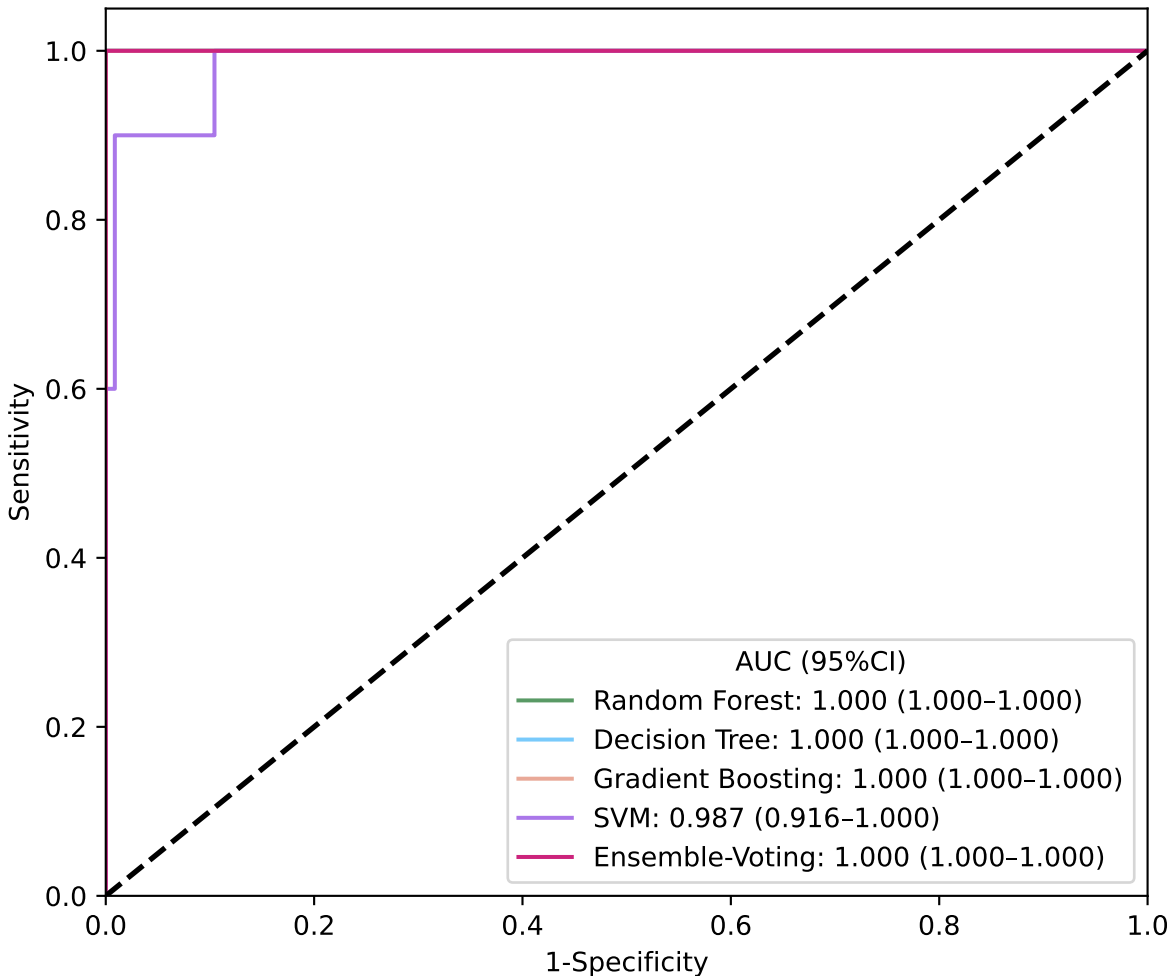

Supplement: Supplementary file 1 [file cancers-17-01974-s001.zip › Supplement Figure S2.pdf]

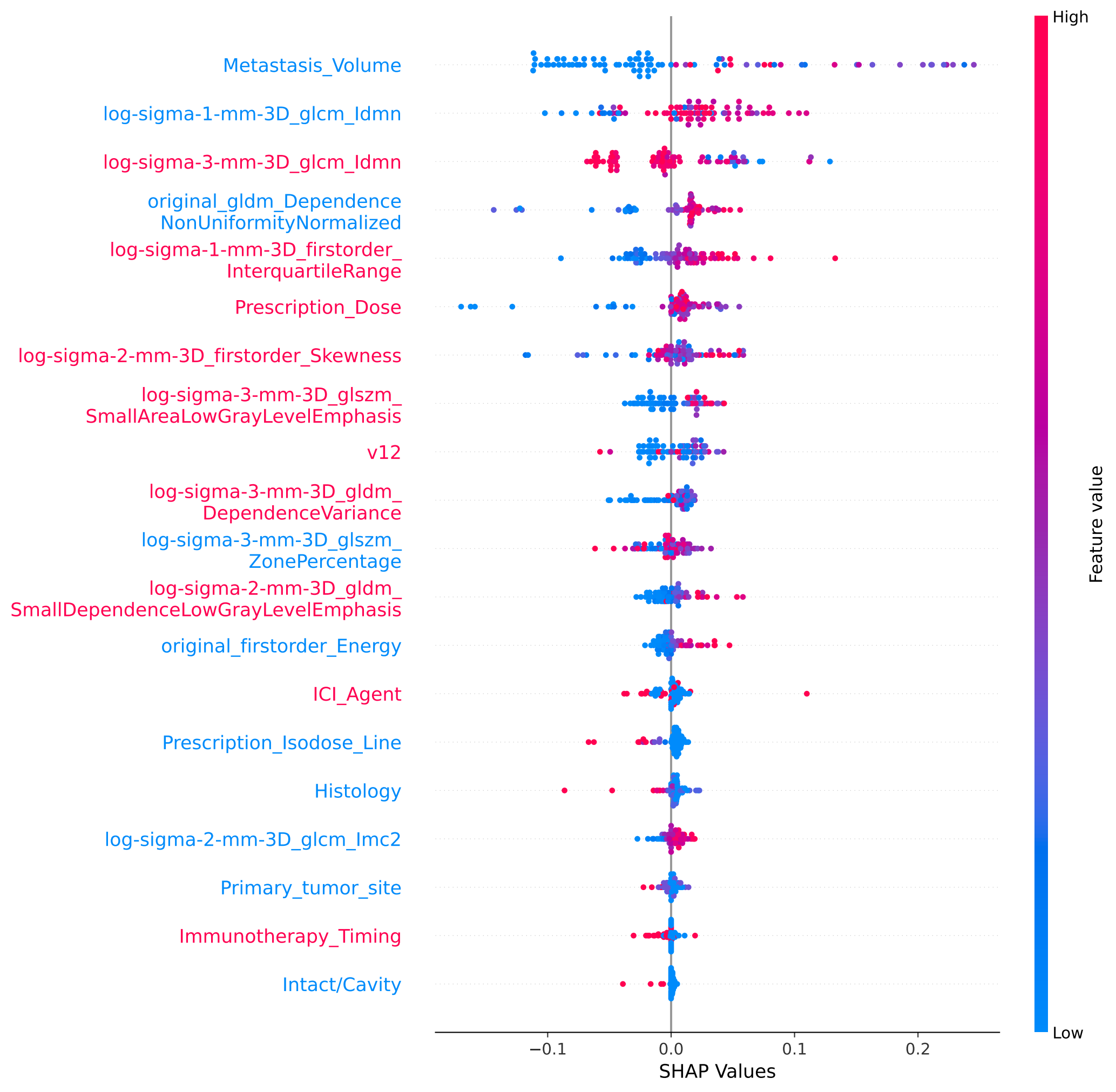

Supplement: Supplementary file 1 [file cancers-17-01974-s001.zip › Supplement Figure S3A.pdf]

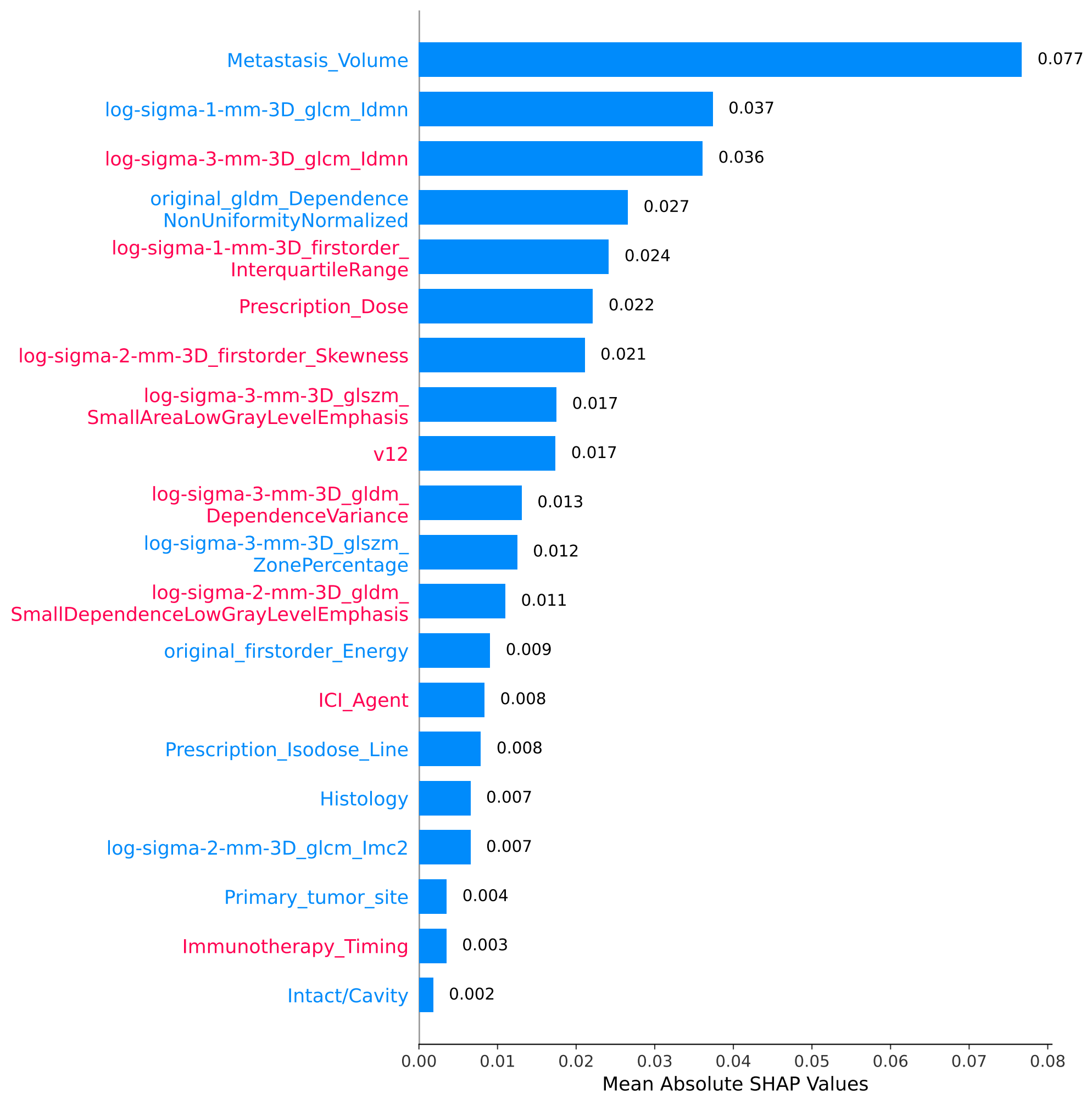

Supplement: Supplementary file 1 [file cancers-17-01974-s001.zip › Supplement Figure S3B.pdf]

Instance 1

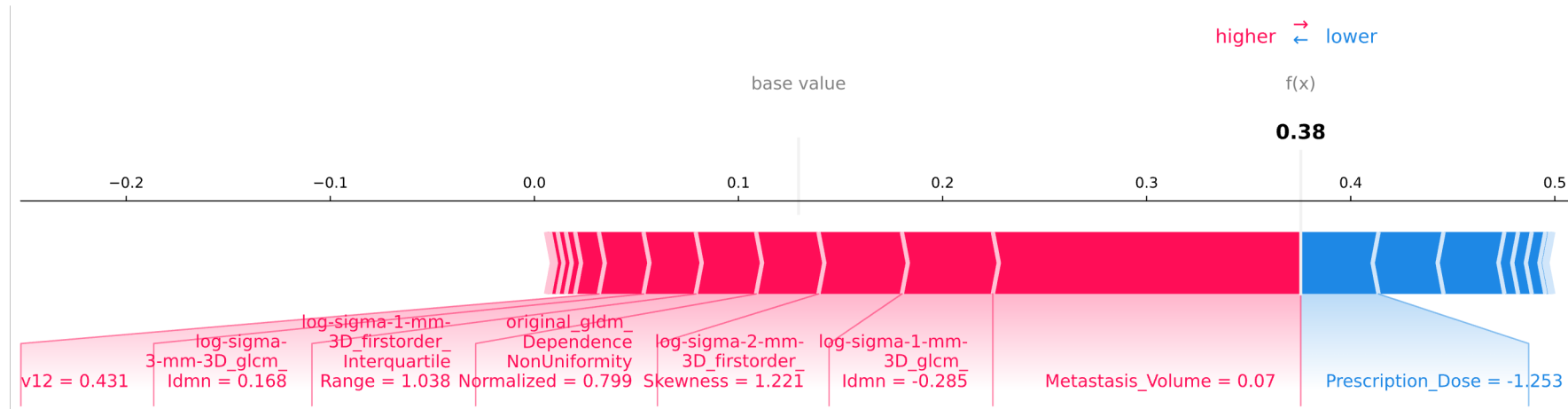

Instance 2

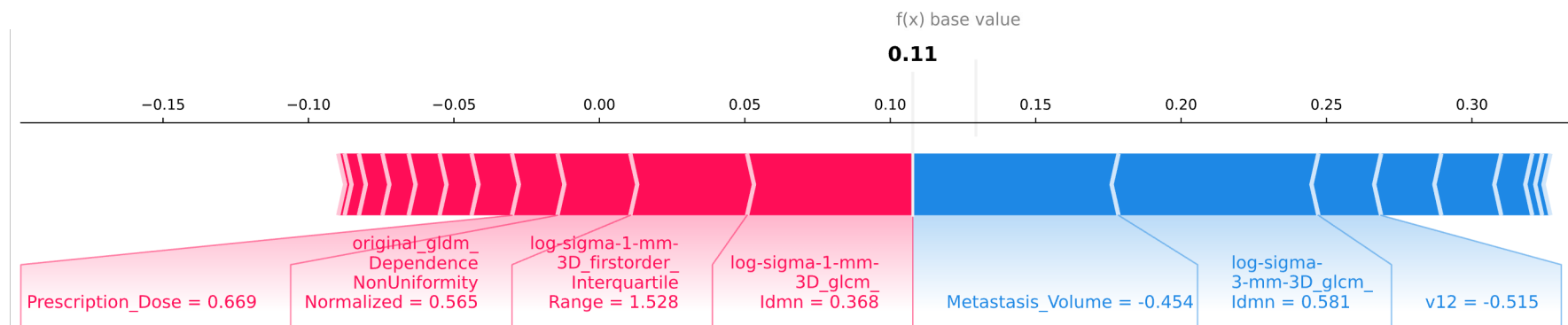

Supplement: Supplementary file 1 [file cancers-17-01974-s001.zip › Supplement Figure S3C.pdf]

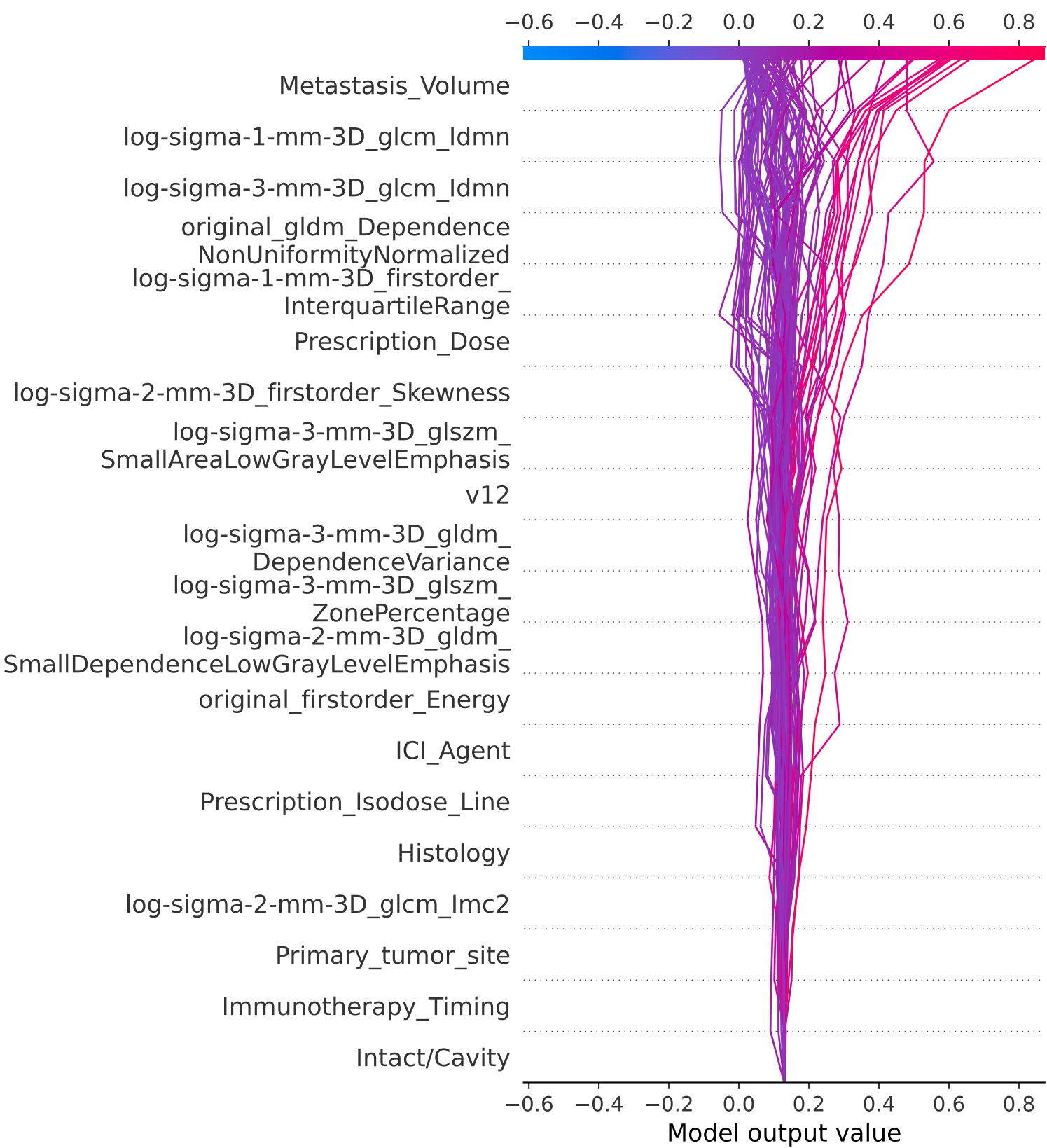

Supplement: Supplementary file 1 [file cancers-17-01974-s001.zip › Supplement Figure S3D.pdf]

LIME: Feature Values

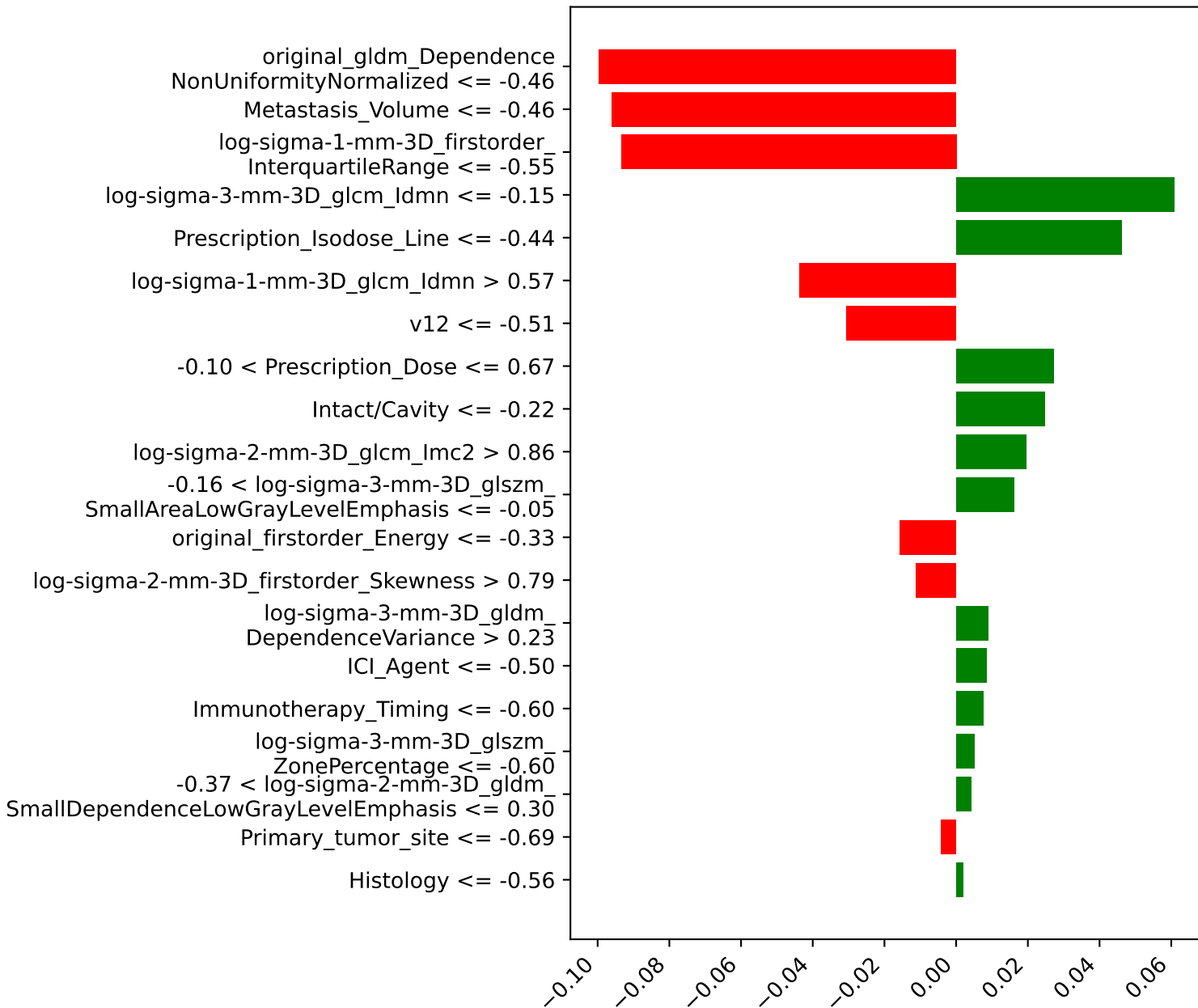

Supplement: Supplementary file 1 [file cancers-17-01974-s001.zip › Supplement Figure S3E.pdf]

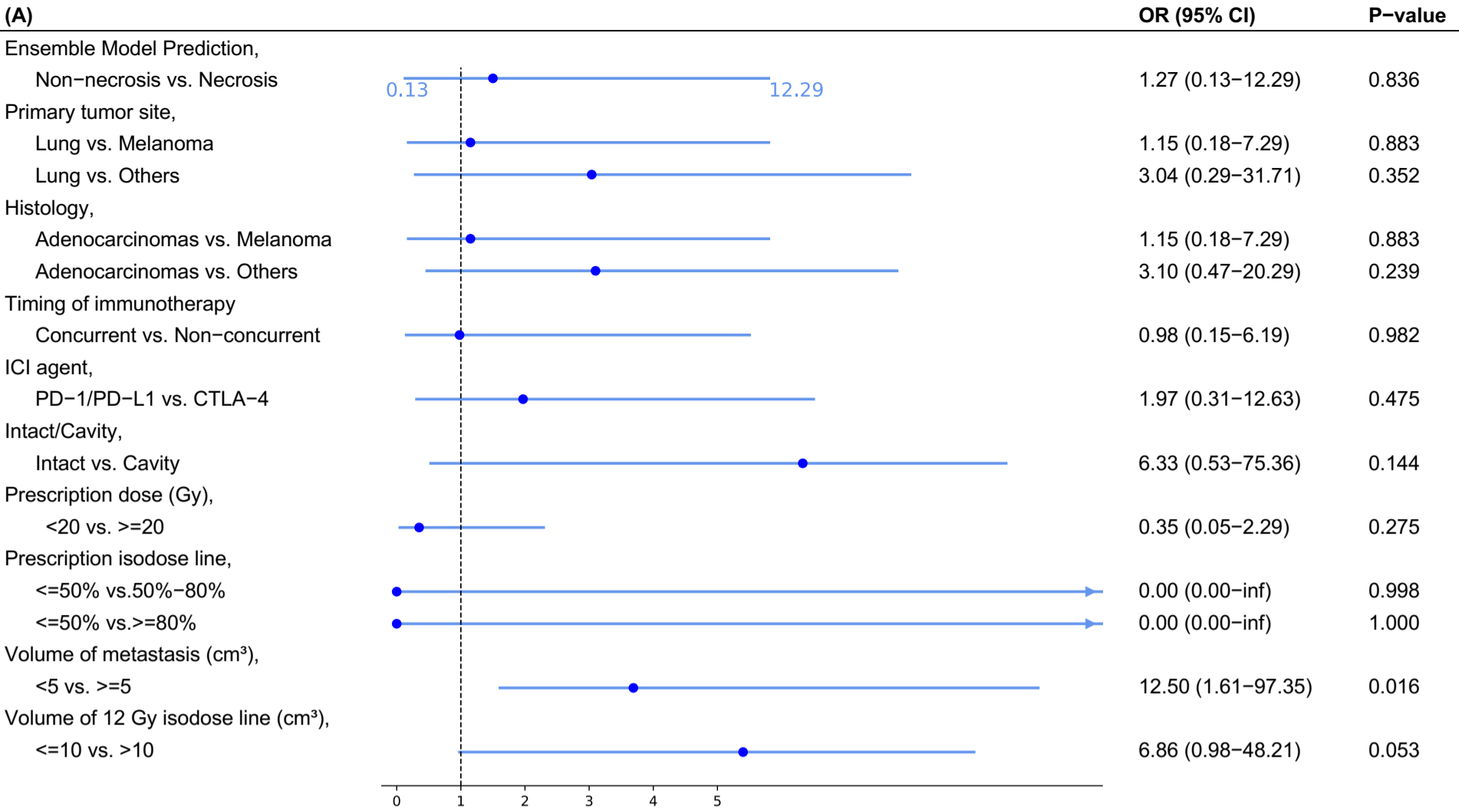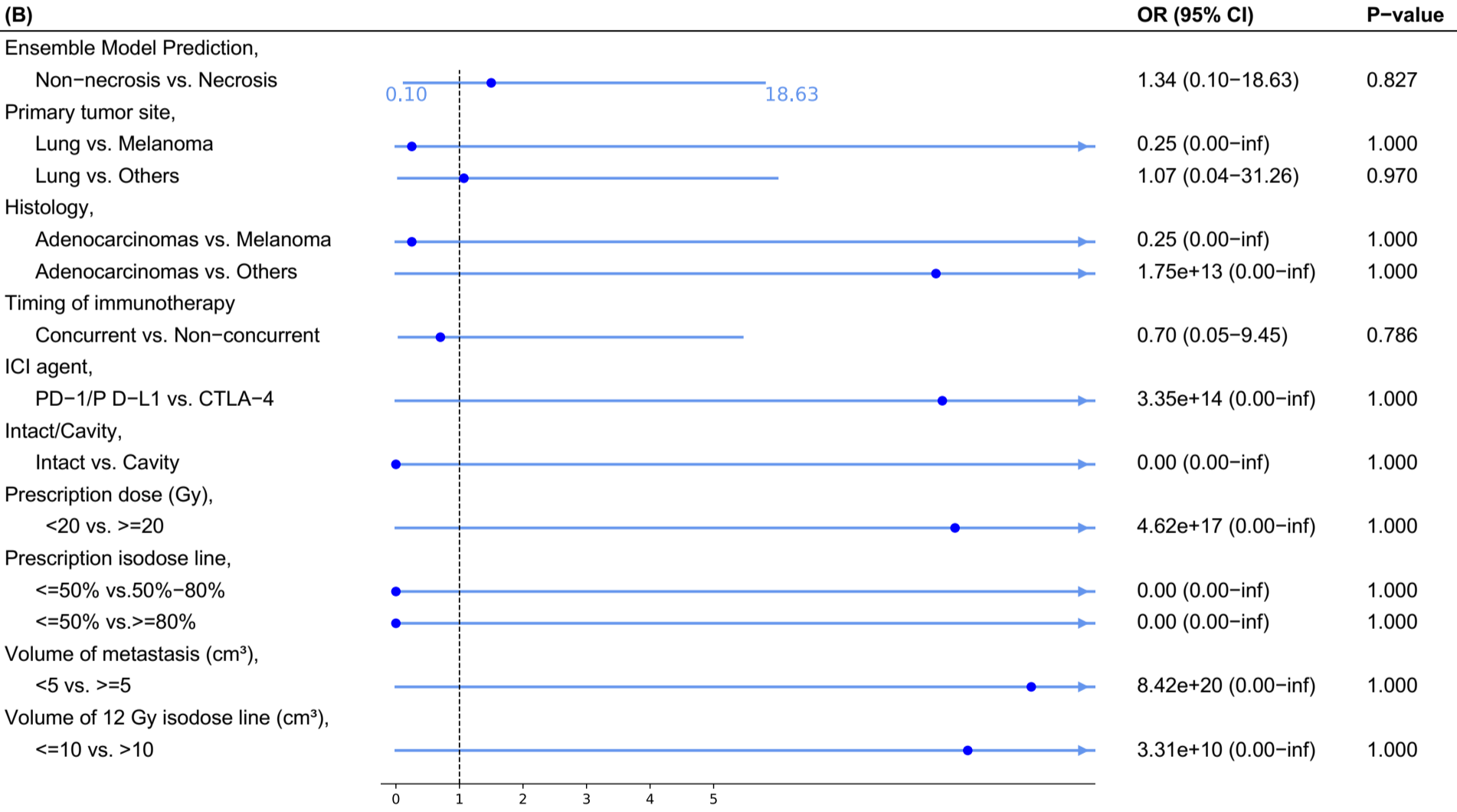

Supplement: Supplementary file 1 [file cancers-17-01974-s001.zip › Supplemental Figure S4.pdf]
